# Identification of Premature Ventricular Cycles of Electrocardiogram Using Discrete Cosine Transform-Teager Energy Operator Model

**DOI:** 10.1155/2015/438569

**Published:** 2015-03-02

**Authors:** Vallem Sharmila, K. Ashoka Reddy

**Affiliations:** ^1^Department of ECE, Kamala Institute of Technology & Science, Huzurabad, Telangana 505468, India; ^2^Department of ECE, Kakatiya Institute of Technology & Science, Warangal, Telangana 506015, India

## Abstract

An algorithm based on the ability of TEO to track the changes in the envelope of ECG signal is proposed for identifying PVCs in ECG. Teager energy is calculated from DCT coefficients of ECG signal. This method can be considered as computationally efficient algorithm when compared with the well-known DCT cepstrum technique. EPE is derived from the teager energy of DCT coefficients in DCT-TEO method and from the cepstrum of DCT coefficients in the existing method. EPE determines the decay rate of the action potential of ECG beat and provides sufficient information to identify the PVC beats in ECG data. EPEs obtained by DCT-TEO and existing DCT cepstrum models are compared. The proposed algorithm has resulted in performance measures like sensitivity of 98–100%, positive predictivity of 100%, and detection error rate of 0.03%, when tested on MIT-BIH database signals consisting of PVC and normal beats. Result analysis reveals that the DCT-TEO algorithm worked well in clear identification of PVCs from normal beats compared to the existing algorithm, even in the presence of artifacts like baseline wander, PLI, and noise with SNR of up to −5 dB.

## 1. Introduction

SA node fires electrical impulses at regular intervals that travel through the conduction pathways of cardiac musculature [1]. Only these excitation impulses allow the contraction and expansion of cardiac muscles which when recorded give ECG consisting of three distinct features referred to as P, QRS, and T waves. Depolarization of left and right atria is summed up as P-wave, tall and narrow QRS component reflects the ventricular depolarization, and T-wave is the result of ventricular repolarisation. When SA node fails to function properly, impulses are generated by the atrial or ventricular musculature, which leads to uncoordinated contraction of ventricular muscles in the heart. In response to the impulses generated in the ventricular muscles, ventricular depolarization occurs earlier which is referred to as PVCs. Energy of a normal beat of ECG data is concentrated within a frequency range of 0–17 Hz in frequency domain, whereas energy of an arrhythmia beat of ECG data is spread over longer distance in time domain which reflects that the energy is compressed to a frequency range of 0–4 Hz, so PVCs can be identified using this energy parameter as a measure. Many algorithms were developed in the literature for identification of PVCs [2] based on rhythm analysis and classification of morphological features [3], where R peak detection is required. O'Dwyer et al. [2] proposed that the signal length and its relation to minimum phase correspondent (MPC) can be used to distinguish normal beats from arrhythmia beats. Mandyam et al. [3] showed that QRS width can be chosen as feature vector for arrhythmia classification. DCT was mostly used in image signal processing applications. Kvedalen [4] documented the basic concepts of DCT and its application for image compression. Many linear operators are available for signal analysis but they are not successful for analysis of nonlinear signals like speech and ECG signals. Maragos and Potamianos [5] introduced a nonlinear energy operator TEO, which was found to be successful in the analysis of nonlinear signals. Kamath [6] suggested that TEO, a Teager-Kaiser (1990) operator, is a higher order discrete energy operator when applied to nonlinear signals, yielding energy as a useful parameter. Kaiser [7] studied the basic concepts of TEO and found that TEO can be used for energy extraction from nonlinear signals and achieved successful classification of arrhythmia beats from normal beats. Basic feature of TEO is that it characterizes the energy of the system that generated the signal other than the energy of the signal [8]. Murthy et al. [9] combined the concepts of DCT and homomorphic filtering (cepstrum) for successful identification of PVCs under noisy conditions up to 10 dB. Homomorphic filtering concepts and its application to ECG signal analysis are reported by Murthy and Prasad [10].

DCT is a trigonometric transformation method [4], having a prominent feature of accumulating all the higher energy coefficients of the signal nearer to the origin which motivated us to use DCT. In this paper, a DCT-TEO modeling based algorithm is proposed to extract the energy of ECG beat for identifying PVCs. Many linear energy operators are available in the literature to extract the energy of linear signals that give energy proportional to the square of the amplitude of the signal. Such operators cannot be used for nonlinear signals as their analysis requires amplitude as well as phase or frequency of the signal. A nonlinear energy operator TEO [5–7] is a simple and efficient model developed by Paul et al. [8] to estimate the energy of the source from which the signal is generated using just only three samples. Specialty of TEO lies in its ability to track the changes occurring in the energy of nonlinear components of signals like ECG and it is best suitable for real time signals. Teager energy extracted from DCT coefficients corresponds to the envelope of the system function of ECG. We found the proposed DCT-TEO algorithm as a simple technique that gives remarkable results in distinguishing the PVC beat from normal beat when compared to the well known DCT-cepstrum algorithm [9]. Decay rate of the envelope of ECG beat was used as a measure to identify PVC beats from normal beats of ECG data. Extraction of the envelope or system function requires low-pass filtering of the DCT coefficients of ECG in cepstrum technique whereas the same envelope can be extracted from three samples of DCT coefficients on applying TEO. The remaining work of this paper is arranged in the following way: proposed DCT and its application are explained in Section 2.1, Teager energy operator in Section 2.2, and results and discussion are in Section 3, followed by conclusions in Section 4.

## 2. Methods

### 2.1. DCT and Application to ECG

Discrete cosine transform (DCT) can linearly transform the data in time domain to the frequency domain by a set of DCT coefficients [4]. DCT represents the signal or data as a sum of cosine functions with different frequencies. For a given ECG signal *x*[*n*] for *n* = 0 … *N* − 1, with equal number of samples around R-peak, *N* point DCT is given as

(1) $$\begin{array}{c} X_{c}\left(k\right)=c_{k}\sqrt{\frac{2}{N}}\overset{N-1}{\underset{n=0}{\sum }}x\left[n\right]\cos\left[\frac{\pi }{N}\left(n+\frac{1}{2}\right)k\right], \end{array}$$

where *X*_*c*_(*k*) represent the DCT coefficients or weights and the constant *c*_*k*_ is

(2) ck=12 for  k=0=1 for  k=1,…,N−1.

The ECG signal *x*[*n*] can be interpreted as the convolution of action potential *e*[*n*] and excitatory function *h*[*n*] in time domain equal multiplication of their corresponding DFTs *E*_*c*_[*k*] and *H*_*c*_[*k*] in frequency domain, expressed by the following equations:

(3) $$\begin{array}{c} \;\text{in}\;\;\text{time}\;\;\text{domain}\;\;x\left[n\right]=e\left[n\right]*h\left[n\right],\\ \;\text{in}\;\;\text{frequency}\;\;\text{domain}\;\;X_{c}\left[k\right]=E_{c}\left[k\right]H_{c}\left[k\right]. \end{array}$$

Therefore the ECG signal can be decomposed into system and excitatory functions using homomorphic or cepstral filtering [3, 10]. Cepstrum technique involves taking the logarithm of the DFT of a given sequence, which converts the multiplication in frequency domain in addition to cepstral domain. By low-pass filtering the cepstral components, system function can be separated from the excitatory function. The system function shows decaying characteristics due to the decaying nature of the DCT coefficients, which is used as distinguishing feature for PVC detection.

### 2.2. Teager Energy Operator (TEO)

Energy of a signal is distributed in the frequency band of the signal. One way of defining the energy of a signal is to consider the squared absolute value of the Fourier transform of the given signal. Let *x*[*n*] be a discrete time signal whose energy *E* is computed as

(4) $$\begin{array}{c} X\left(k\right)=\text{DFT}\left(x\left[n\right]\right)=\frac{1}{N}\overset{N-1}{\underset{n=0}{\sum }}x\left[n\right]e^{-j2\pi nk/N}. \end{array}$$

Energy of the signal *x*[*n*] is given as

(5) $$\begin{array}{c} E={\left|X(k)\right|}^{2}. \end{array}$$

**Figure 1:**
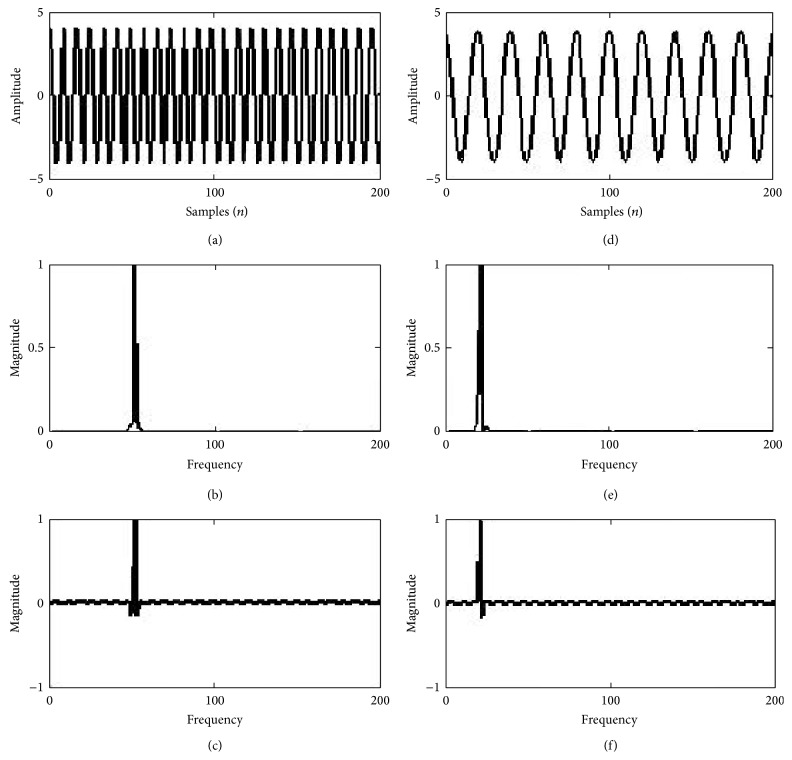
50 Hz sine wave in trace (a); energy using FFT in trace (b); TE in trace (c); 20 Hz cosine wave in trace (d); energy using FFT in trace (e); and TE in trace (f).

**Figure 2:**
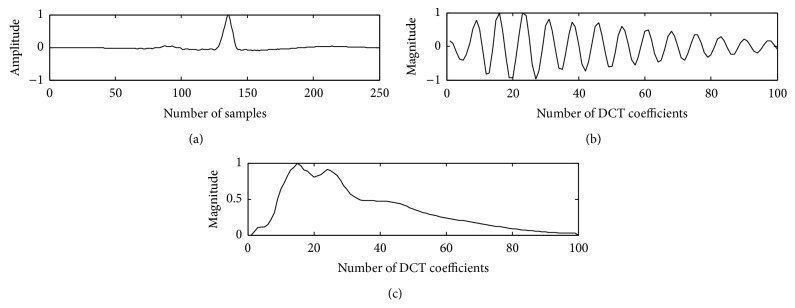
Normal beat in trace (a); DCT coefficients in trace (b); and Teager energy in trace (c).

From the above equations it can be observed that two signals of the same amplitude and different frequencies will exhibit the same energy as the energy is directly proportional to the square of the amplitude of the signal. This is illustrated with an example in Figure 2 by considering a sinusoidal signal of amplitude 2 v and 50 Hz frequency and a second sine wave of amplitude 2 v and 25 Hz with the same sampling frequency *fs* = 400 Hz is exhibiting the same energy of 4.09*e* + 005 units. But according to Kaiser, energy required to generate a sine wave varies as a function of both amplitude and frequency from the study of second order differential equation which motivated him to derive Teager energy operator (TEO). TEO concept implies that the energy required to generate a signal is directly proportional to the frequency of the signal. It was observed that the energy required to generate a 50 Hz signal is 7.9 units and energy required to generate a low frequency signal is 1.5 units using TEO from example illustrated in Figure 1.

TEO concept was derived from the solution of a second order differential equation which describes the functioning of a nonlinear signal. TEO model is used to extract the instantaneous energy of the source that generated the signal. ECG signal is generated from SA node due to the depolarization and repolarization of atria and ventricles in the cardiac muscles of the heart. As per Kaiser it can be represented by a second order differential equation

(6) $$\begin{array}{c} \frac{d^{2}x}{dt^{2}}+\frac{k}{m}x=0, \end{array}$$

where *x*(*t*) is the generated ECG signal with respect to time, *m* is the mass of the heart, and *k* is a constant. Solution of the differential equation is the rhythmic heart beat given as

(7) $$\begin{array}{c} x\left(t\right)=A\cos\left(\omega t+\varphi \right), \end{array}$$

where *A* is the amplitude with initial phase *φ*.

Similarly *x*[*n*] is the generated ECG signal in discrete domain represented as

(8) $$\begin{array}{c} x\left[n\right]=A\cos\left(\Omega n+\Phi \right), \end{array}$$

where *Ω* is the digital frequency in radians/sample given as

(9) $$\begin{array}{c} \Omega =\frac{2\pi f}{fs}, \end{array}$$

where *f* is the analog frequency, *fs* is the sampling frequency, and Φ is the initial phase. *Ω*, *f*, and Φ are the three unknown parameters for which the solution is given by Kaiser using three samples *x*[*n*], *x*[*n* − 1], and *x*[*n* + 1]. The energy at any given instant of time *n* is given as

(10) En=xn2−x[n−1]x[n+1]=A2sin2(Ω)EnA2Ω2 for  small  Ω.

The above energy equation works well with the constraint of *Ω* to be positive and less than one quarter of the sampling frequency for which the approximation error is less than 11%. TEO is defined from the above concept as the squared product of instantaneous amplitude and corresponding frequency.

### 2.3. Proposed Method Based on DCT-TEO

ECG signal can be considered as modulation of action potential (base band signal) generated from the SA node with the excitation generated in the cardiac muscles. Teager energy evaluated for an ECG signal corresponds to the energy of the action potential generated by the SA node in the heart. It can be observed that TEO can be used to track the modulation energy (envelope) and identify the amplitude and frequency at any instant of time. SA node fires the impulses at a rate of 60–100 beats/min for a healthy person, where, as if SA node fails, its function is taken up by pacemaker cells which fires the impulses at a lower rate of 40–60 beats/min. Energy measured by TEO model reflects the disturbances in the impulse generation and conduction path. Keeping in view that TEO is sensitive to noise, TEO is applied on DCT coefficients of ECG beat, as DCT improves the signal to noise ratio. The algorithm for estimation of the envelope using TEO is given below.

(1) Compute DCT coefficients of the ECG signal *x*[*n*], that is, *X*_*c*_[*k*].
(2) Consider three samples: *X*_*c*_[*k*], *X*_*c*_[*k* − 1], and *X*_*c*_[*k* + 1].
(3) Estimate the energy of DCT coefficients using TEO from the expression in  (10).
(4) Calculate the decay rate defined as the number of DCT coefficients in which 90% of the energy is packed.
(5) Compare the decay rates of both DCT-cepstrum technique and DCT-TEO model.
(6) Identify the PVC beats from normal beats of ECG record.

## 3. Results

### 3.1. PVC Detection

**Figure 3:**
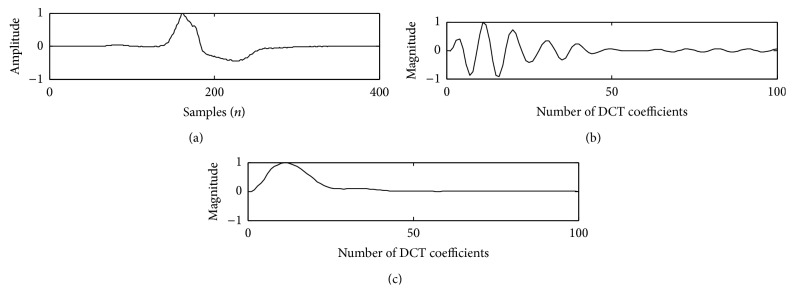
PVC beat in trace (a); DCT coefficients in trace (b); and Teager energy in trace (c).

The proposed method is tested on the ECG data records and compared with the existing method of DCT-cepstrum. Envelopes extracted from DCT coefficients using nonlinear Teager energy operator for a PVC beat and normal beat with the proposed method are shown in Figures 2 and 3.

It can be seen that the envelope of PVC beat decays at a faster rate due to the wider QRS complexes in time domain compared to normal beats. Only first 150 coefficients can be considered for further analysis since the DCT coefficients decay to zero beyond 150 samples. Energy packing efficiency (EPE) denoted by *ε*_*i*_ is considered as a quantitative measure to identify PVC beats. Energy is packed in first *i* DCT coefficients, as DCT brings all the high frequency energy components nearer to the origin. Energy *ε*_*i*_ being packed in the *i*th coefficient is calculated as

(11) $$\begin{array}{c} \varepsilon_{i}=\frac{\sum_{0}^{i}E_{i}^{2}}{\sum_{0}^{N-1}E_{i}^{2}}. \end{array}$$

**Figure 4:**
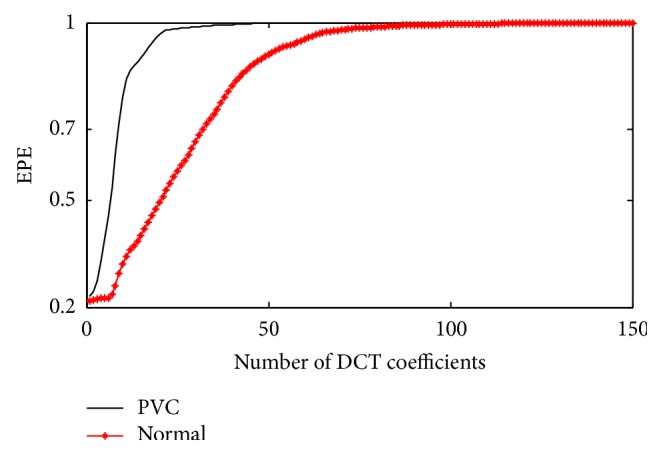
EPE curves for PVC and normal beats with DCT–TEO.

It is a measure used to calculate a number of DCT coefficients in which 90% of the energy is packed. Hence *ε*_*i*_ = 0.9 is considered as distinguishing factor for arrhythmia beats and normal beats. EPE computed with the proposed DCT-TEO method differs for PVC beat and normal beat as shown in Figure 4.

### 3.2. Comparison with DCT-Cepstrum

**Figure 5:**
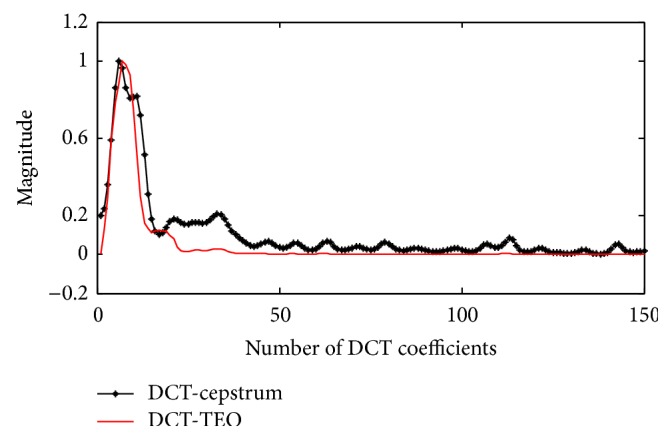
Extracted envelope for PVC data using DCT-cepstrum and DCT-TEO.

The proposed method is compared with the estimation of envelope using DCT-cepstrum which requires more logic than the DCT-TEO method. In the DCT-cepstrum technique, cepstral analysis of DCT coefficients was used to identify the PVCs. Cepstrum can be decomposed into system and excitatory functions using cepstral filtering. Envelopes extracted using both the models are shown in Figure 5 from which it can be clearly seen that, unlike DCT-cepstrum method, the proposed TEO based method results in a simple impulsive like envelope.

**Figure 6:**
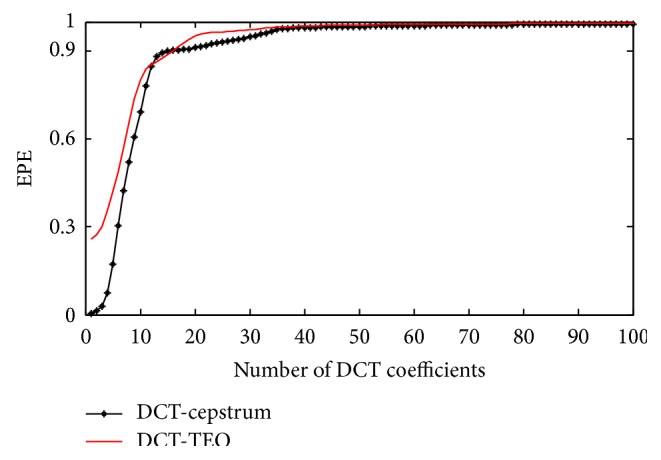
EPE curves evaluated for PVC beat using DCT-cepstrum and DCT-TEO.

EPE curves drawn for PVC beats using both the methods are shown in Figure 6. With DCT-cepstrum technique, *ε*_*i*_ = 0.9 is reached within the first 10–15 coefficients for a PVC beat and exceeds 34–48 coefficients for a normal beat. In case of proposed DCT-TEO method, *ε*_*i*_ = 0.9 is reached within 5–11 coefficients for a PVC beat whereas it exceeds 29–39 coefficients for a normal beat after which the energy decays to zero. TEO is able to identify the PVC beat with few DCT coefficients whereas DCT-cepstrum model is identifying the PVC beat with more number of DCT coefficients which imply that more computations are required by DCT-cepstrum model than DCT-TEO model in the PVC beat identification. The number of DCT coefficients, which represent 90% of the energy (*ε*_*i*_ = 0.9) are identified by computing equation (11) for different records of arrhythmia data and details are presented in Table 1. Different performance measures like sensitivity, positive predictivity, and detection error rate calculated with DCT-TEO model are shown in Table 2. Sensitivity is the ability to detect the PVC beats, calculated based on the decay rate of the DCT coefficients. Positive predictivity is the ability to predict the PVC beats and a low detection error rate indicates the efficiency of the algorithm in identifying PVCs.

### 3.3. Artifacts and Noise Sensitivity

**Figure 7:**
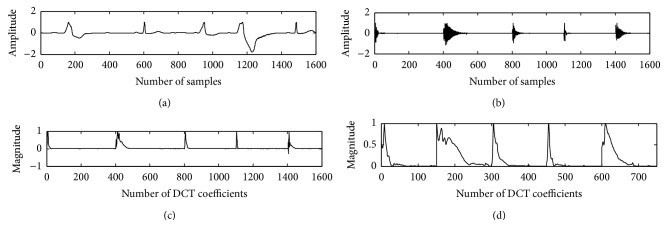
Record 208 in trace (a); DCT coefficients in trace (b); DCT-cepstrum in trace (c); and DCT-TEO in trace (d).

**Figure 8:**
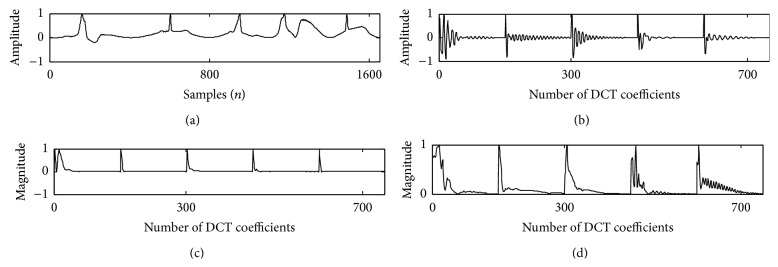
Record 208 with baseline wander noise in trace (a); DCT coefficients in trace (b); DCT-cepstrum in trace (c); and DCT-TEO in trace (d).

**Figure 9 fig9:**
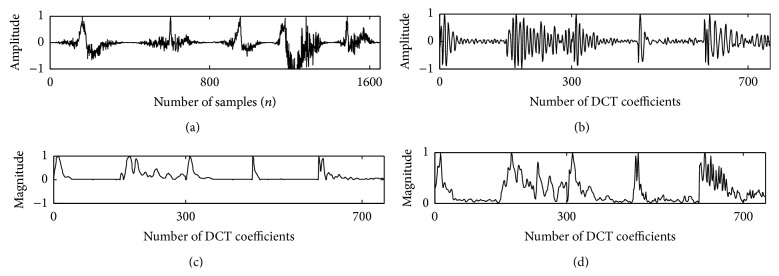
208 with Gaussian noise in trace (a); DCT coefficients in trace (b); DCT-cepstrum in trace (c); and DCT-TEO in trace (d).

**Figure 10:**
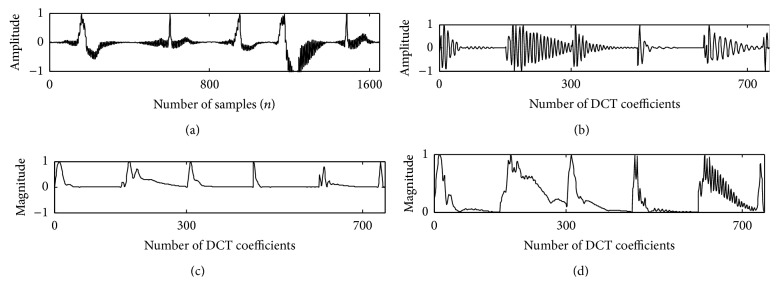
Record 208 with PLI noise in trace (a); DCT coefficients in trace (b); DCT-cepstrum in trace (c); and DCT-TEO in trace (d).

**Figure 11:**
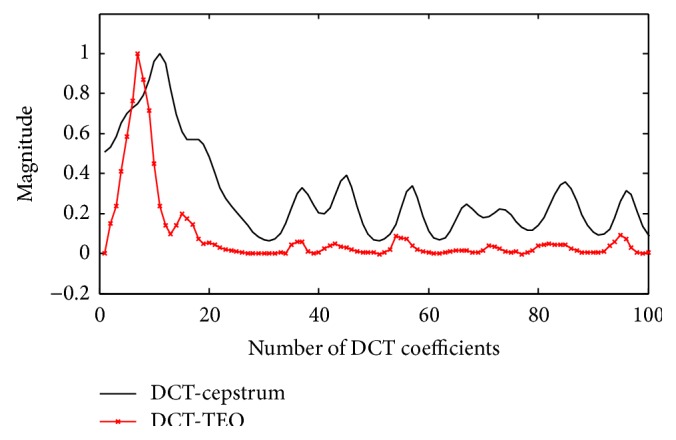
Envelopes extracted for noisy PVC for SNR = −5 dB using (a) DCT-cepstrum and (b) DCT-TEO.

Teager energy of the DCT coefficients reveals a peak corresponding to the envelope of system function. This peak decays at a faster rate of 5–11 coefficients for a PVC beat, which reflects a wider QRS complex in time domain, whereas the peak of a normal beat decays within 29–39 coefficients which reflects a narrow QRS complex as shown in Figure 7. 50/60 Hz PLI noise and low frequency (0.1 Hz) baseline wander noise alters the shape of ECG waveform which makes it difficult to analyze the cardiac pathology of a person. PLI noise interferes with the recording of ECG data through the power cable. Baseline wander noise occurs due to the muscle movement of the patient while recording the ECG data which shifts the isoelectric line of ECG signal. Identification of PVC beat from normal beat, even in the presence of the above artifacts, is made possible by applying TEO compared to DCT-cepstrum. Muscle artifact affected ECG beat was obtained by adding zero mean white Gaussian noise. The unit variance noise samples when multiplied with a proper scaling factor give required SNR. The algorithm when applied on different records of MIT-BIH database, consisting of both normal and PVC beats, in the presence of PLI, baseline wander artifacts, and a −5 dB Gaussian noise is shown in Figures 8 [Fig fig9]–10. TEO can successfully extract the envelope of action potential for noisy records of normal and PVC beats up to SNR of 5 dB. EPEs evaluated for noisy beats show a similar graph to that of ECG beats without addition of noise. It can be clearly seen from the results that, for a PVC beat, the envelope of DCT-TEO is decaying at a faster rate clearly giving only one peak. This property is very much important in identifying PVC beats in highly noise environment. The noise sensitivity test was carried out up to SNR of −5 dB. The extracted envelope is shown in Figure 11. The results establish the fact that the envelopes extracted by TEO are tending to exhibit a monotonically decreasing envelope, which makes it to clearly identify the PVCs in even high noise environment.

**Figure 12:**
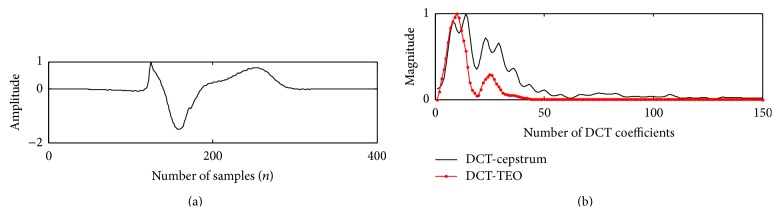
Paced beat in trace (a); envelope of DCT-cepstrum and DCT-TEO in trace (b).

*Application of Proposed Method to Paced Beats*. When SA node fails to fire the impulses in the heart artificial pacing is required for conduction of the impulses in the heart. Electrical pulses conduct through left or right bundle branches from atrial musculature to the myocardium in the ventricles. Blocks in these conduction pathways give rise to left bundle branch block (LBBB) or right bundle branch block (RBBB). In LBBB right ventricle depolarizes prior to left ventricle resulting in splitted QRS complex with the second half of the complex being wider than the first due to lack of synchronization between left and right ventricles. Paced beat obtained at the right ventricle is the same as PVC of LBBB morphology and at the left ventricle is equal to the PVC of RBBB morphology. Envelope obtained for paced beats as shown in Figure 12 enables distinguishing PVC beat from paced and bundle branch blocks. Envelopes estimated by DCT-cepstrum can only approximate as the phase information is lost in the DCT coefficients where the envelope obtained with nonlinear TEO is accurate.

## 4. Conclusions

TEO has an attractive feature of following the instantaneous changes occurring in the energy of nonlinear signals which motivated us to use this operator for the analysis of nonlinear ECG data. So an attempt has been made to use TEO for extracting the energy of the envelope of ECG using just only three samples to identify PVCs from normal beats based on the decay rate of the envelope of DCT coefficients. DCT has the important characteristic of expressing the signal as sum of cosine (even) functions with different frequencies and concentrating the energy in only a few low frequency components nearer to the origin. So initially the ECG beat is transformed to frequency domain using DCT. Envelope of the ECG beat is obtained from the DCT coefficients using nonlinear Teager energy operator and compared with a known technique of cepstral filtering [8]. The envelope of ECG beat decays at a faster rate for a PVC beat which reflects wider QRS complex of PVC beat compared to normal beat. The rate of decay of the envelope is used as identifying factor for PVC beats from normal beats. For PVCs this number varies from 10 to 15 coefficients with cepstrum model and from 5 to 11 coefficients using TEO model whereas for a normal beat it varies from 34 to 48 coefficients with cepstrum model and from 29 to 39 coefficients with TEO model. TEO model is found efficient in identifying PVC beats present in different types of arrhythmia records with lesser number of DCT coefficients even in the presence of artifacts like baseline wander noise, PLI noise, and a highly noisy environment with signal to noise ratio of up to −5 dB, whereas cepstrum model is able to identify the PVCs only in the presence of Gaussian noise with large number of DCT coefficients. Statistical measures like sensitivity, predictivity, and detection error rate are also calculated for the proposed algorithm as shown in Table 2. Further, the envelopes obtained using proposed DCT-TEO method for paced beats, LBBB, and RBBB clearly distinguished PVC beat from paced and bundle branch blocks. The proposed algorithm can be extended for ECG signal enhancement and detection of various arrhythmias. Full forms and acronyms are shown in Table 3.

## Figures and Tables

**Table 1:** Number of DCT coefficients for *ε*_*i*_ = 0.9 for various ECG records.

ECG data record.	Number of DCT coefficients for which *ε*_*i*_ = 0.9 is reached	
	Cepstrum method	Proposed TEO method
208 (normal beat)	34–48	29–39
208 (PVC beat)	10–15	5–11
111 (LBBB)	35–40	24–28
124 (RBBB)	35–37	24–28
217 (paced beat)	13–18	13–16

**Table 2:** Identification of PVC beat from MIT-BIH database, with proposed algorithm.

PVC record	Actual number of PVCs	FP	FN	Failed detection	Se (%)	Detection error rate (%)	+*P*%
**119**	444	0	1	1.0	98	0.03	100
**124**	47	0	0	0	100	0.0	100
**200**	826	0	1	1.0	99	.03	100
**208**	992	0	1	1.0	99	.016	100
**221**	396	0	0	0.0	100	0.0	100
**210**	194	0	0	0.0	100	0.0	100

**Table 3:** List of acronyms.

S. no.	Full form	Acronym
1	Electrocardiogram	ECG
2	Sinoatrial node	SA node
3	Premature ventricular cycle	PVC
4	Discrete cosine transform	DCT
5	Teager energy operator	TEO
6	Energy packing efficiency	EPE
7	Power line interference	PLI
